# Engaging T cells for cleanup

**DOI:** 10.3389/fimmu.2025.1551424

**Published:** 2025-05-06

**Authors:** Roman V. Mungalov, Natalia V. Mushenkova, Dmitriy M. Chudakov, Maria A. Turchaninova

**Affiliations:** ^1^ Institute of Translational Medicine, Pirogov Russian National Research Medical University, Moscow, Russia; ^2^ Genomics of Adaptive Immunity Department, Institute of Bioorganic Chemistry, Moscow, Russia; ^3^ Faculty of Biology and Biotechnology, Higher School of Economics, Moscow, Russia; ^4^ Unicorn Capital Partners, Moscow, Russia; ^5^ Center for Molecular and Cellular Biology, Moscow, Russia; ^6^ Department of Molecular Medicine, Central European Institute of Technology, Brno, Czechia; ^7^ Abu Dhabi Stem Cell Center, Al Muntazah, United Arab Emirates

**Keywords:** T-cell engager, soluble TCR, immunotherapy, gene engineering, autoimmunity

## Abstract

T-cell engagers represent a transformative approach to cancer immunotherapy leveraging bispecific and multispecific antibody constructs to redirect T-cell cytotoxicity toward malignant cells. These molecules bridge T cells and tumor cells by simultaneously binding CD3 on T cells and tumor-associated antigens on cancer cells, thereby enabling precise immune targeting even in immunologically “cold” tumors. Recent advancements include conditional T-cell engagers activated by tumor microenvironment proteases to minimize off-tumor toxicity as well as T-cell receptor–based engagers targeting intracellular antigens via MHC presentation. Clinical successes, such as Kimmtrak in metastatic uveal melanoma, underscore good potential of these modalities, while challenges persist in the management of cytokine release syndrome, neurotoxicity, and tumor resistance. Emerging multispecific engagers are aimed at enhancing efficacy via incorporation of costimulatory signals, thus offering a promising trajectory for next-generation immunotherapies. T-cell engagers are also gaining attention in the treatment of autoimmune disorders, where they can be designed to selectively modulate pathogenic immune responses. By targeting autoreactive T or B cells, T-cell engagers hold promise for restoring immune tolerance in such conditions as HLA-B*27–associated autoimmunity subtypes, multiple sclerosis, rheumatoid arthritis, and type 1 diabetes mellitus. Engineering strategies that incorporate inhibitory receptors or tissue-specific antigens may further refine T-cell engagers’ therapeutic potential in autoimmunity, by minimizing systemic immunosuppression while preserving immune homeostasis.

## Highlights

- There are two main approaches to T-cell–based immunotherapy: HLA-restricted and HLA-nonrestricted immunotherapy, which are mediated by naturally occurring or genetically engineered T cells to target cancer antigens.
- T-cell engagers (TCEs) are bimodal molecules targeting a universal (e.g., CD3 or T-cell receptor [TCR]) or subtype-specific (e.g., CD8) surface molecule on the T cell and a specific protein or nonprotein molecule or peptide–MHC complex on the surface of a target cell.
- BiTEs (bispecific T-cell engagers) can be based on antibodies targeted at cell surface tumor-associated antigens or peptide–MHC complexes (TCR-mimic antibodies).
- BiTEs can also be based on soluble TCRs with enhanced affinity, representing promising immunotherapeutic candidates naturally selected to recognize peptide–MHC complexes.
- TCEs may become the most powerful tools for specific elimination of transformed and autoantigen-specific cells and informed modulation of immune responses, with accumulated knowledge facilitating rapid development of safe and effective TCE-based interventions (**Table 1**).

**Table 1:** T-cell–engaging arsenal.

Receptor Type	Mechanism	Example	Ref.
scFv	Binding to CD3 on T cell and TAA on tumor cell, followed by activating T cell to eliminate tumor cells	Blincyto	(1)
IgG-like antibody	Binding to CD3 on T cell and TAA on tumor cell as well as FcγR on innate-immunity cells, followed by activating T cells and macrophages to eliminate tumor cells and extend engagers’ half-life	B7-H6/CD3	(2)
Soluble TCR	Binding to CD3 on T cell and pMHC on tumor cell, thus redirecting T cell to target tumor cell	Kimmtrak	(3)
Soluble pMHC	Binding to TCR on T cell, followed by killing of autoreactive T cell via ADC	MHC-MMAF (monomethyl auristatin F)	(4)

## Introduction

### The enhancing strategy

**Figure 1:**
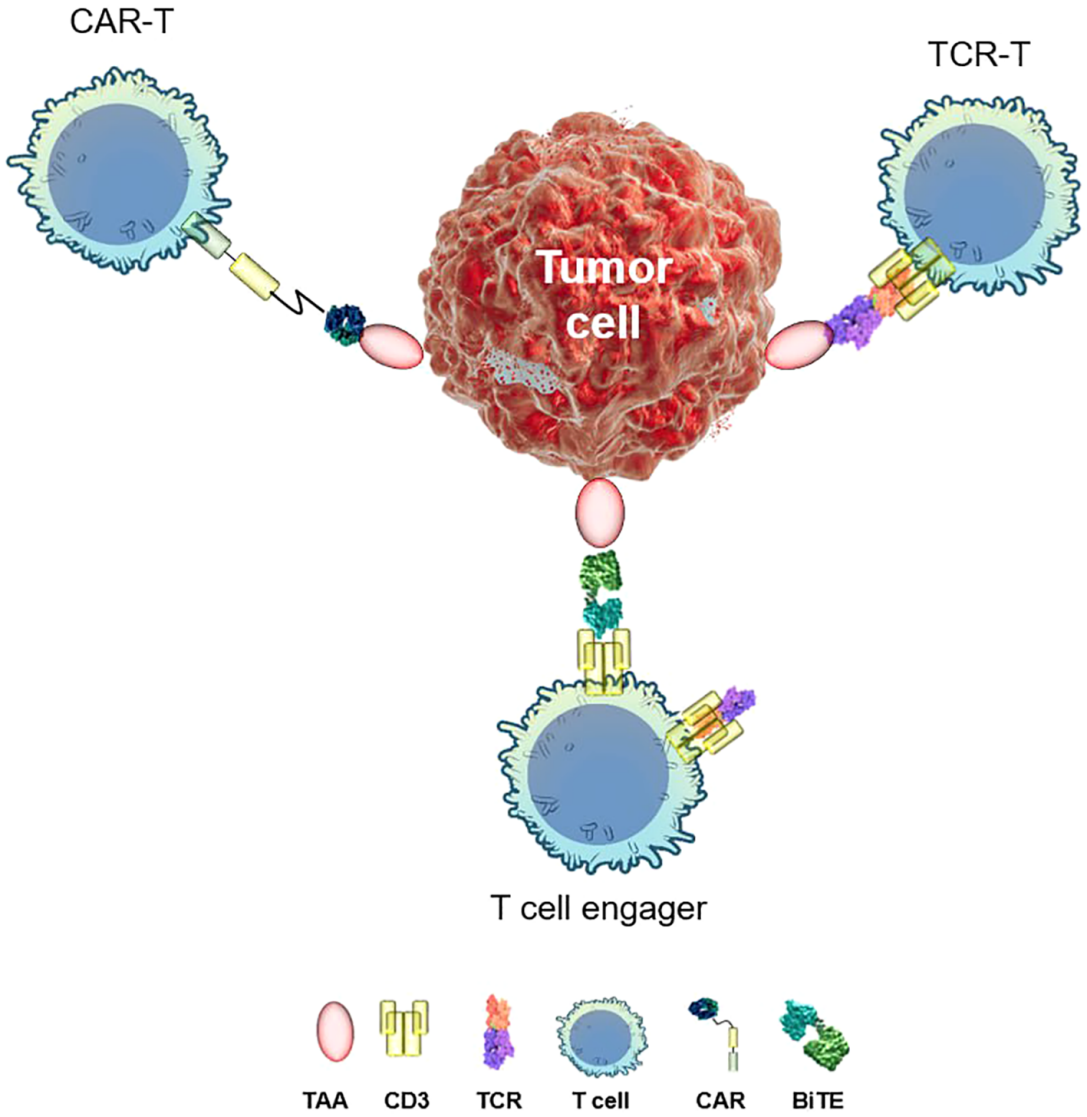
Distinct targeted immunotherapy approaches. TAA, tumor-associated antigen; TCR, T-cell receptor; CD3, cluster of differentiation 3; CAR, chimeric antigen receptor; BiTE, bispecific T-cell engager.

Most of immunotherapeutic approaches employ the body’s own T lymphocytes to combat disease (**Figure 1**). Broadly, there are *two strategies* for reprogramming T cells to target specific threats. The first involves optimizing polyclonal T cells for precise recognition and elimination of tumor cells. Early adoptive cell transfer protocols (5) relied on expansion of the tumor-infiltrating lymphocyte (TIL) population *ex vivo* before re-infusing them into the patient. TILs offer key advantages, including efficient tumor site trafficking and inherent polyclonality, which enhances their ability to recognize diverse tumor antigens (6). A landmark advancement in TIL-based cancer therapy came in 2024 with the FDA approval of Amtagvi (lifileucel) for patients with advanced melanoma who had previously undergone treatment with PD-1 inhibitors (7). Amtagvi has shown promising results in heavily pretreated patients, including those whose cancer has progressed after treatment with immune-checkpoint inhibitors and BRAF inhibitors. The overall response rate (ORR) has been 31%, median duration of response >27 months, and median overall survival over 14 months (7). This is a significant improvement over standard chemotherapy, which has an ORR of 4–12% and a median overall survival of approximately 6 months. This milestone underscores therapeutic potential of TILs in overcoming resistance mechanisms and improving patient outcomes.

As of early 2025, 56 active TIL trials in melanoma were in progress, including two phase 3 trials. The completion of these trials is expected in 2027 and 2028. TILs have also shown promise in the treatment of non–small cell lung cancer that is resistant to immune-checkpoint inhibitor therapy and chemotherapy, including patients with PD-L1–negative and low-TMB tumors (8). A phase 2 trial in non–small cell lung cancer is ongoing, with results expected in 2030. The improvement of TIL technology may be achieved by enrichment with tumor-specific clonotypes (9) and cultivation methods to diminish exhaustion (10). There are concerns about potential cardiotoxic effects of TIL therapy. Hypotension-requiring treatment in approximately 32.6% of patients receiving TILs has been reported, just as cases of atrial fibrillation (14%) and troponin upregulation (2.3%) (11). Toxicity associated with on-target off-tumor recognition and IL-2 infusions has also been reported (12).

Current developments are targeted toward genetic engineering approaches to the adoptive transfer of *ex vivo*–modified immune cells (13). *In vivo* transduction approaches are evolving concurrently, and nearly a dozen products may enter clinical trials by 2026 (14). A chimeric antigen receptor (CAR) is a transmembrane receptor molecule that combines antigenic specificity of an antibody with the effector function of a T cell (15). Several generations of CARs have been created to date and differ in the design of the intracellular signaling part. Since 2017, seven chimeric antigen receptor T cell (CAR-T) products that target CD19 and BCMA antigens have gained FDA approval and shown breakthrough efficacy in hematological cancers (16). Their current limitations include safety concerns due to the high rate of grade 3–4 cytokine release syndrome (CRS) and evidence of recurrence risk. Long-term follow-up studies have shown some risk (3.6%) of secondary primary malignant tumors after commercial CAR-T therapy; these rates are comparable to those observed with chemo- and radiotherapy and remain lower than those reported for stem cell transplantation (17). Similarly to antibodies and antibody–drug conjugates (ADCs), off-tumor toxicity remains a major limitation, significantly restricting clinical applications. In solid tumors, the microenvironment and tumor heterogeneity pose additional efficacy barriers (18, 19). Nonetheless, some clinical trials dealing with solid tumors show promising results. In stage IV pancreatic ductal adenocarcinoma (PDAC), the next-generation CAR-T has shown significant clinical results with up to a 40% reduction in tumor size after the initial cycle (20). Currently approved CAR-T therapeutics are hard to produce and expensive, and the need for patient preconditioning limits their therapeutic niche. Emerging *in vivo* CAR-T approaches (13, 21) represent a significant advancement in this field.

A critical challenge in targeted immunotherapies is antigen loss due to tumor heterogeneity and immune editing, which compromise therapeutic efficacy. This limitation has spurred interest in combinatorial approaches, such as using oncolytic viruses (OVs) to induce *de novo* expression of CAR-targetable antigens within tumors (22, 23). The convergence of CAR-T therapy and OVs holds promise for overcoming antigen variability in solid tumors because OVs can remodel the tumor microenvironment (TME) to enhance CAR-T activity (24, 25). Innovations such as locoregional delivery (e.g., intraventricular or intrapleural administration) and multiantigen targeting (e.g., bispecific CARs) showed improved tumor infiltration and reduced antigen escape in recent trials (26, 27). Furthermore, such strategies as cytokine-secreting CARs and synthetic biology tools such as switchable receptors are aimed at counteracting immunosuppressive TME barriers, e.g., hypoxia, nutrient scarcity, and regulatory immune cells (28).

The use of T-cell receptor (TCR)-based recognition helps to expand the repertoire of targetable antigens because T-cell receptor T cells (TCR-T) can recognize epitopes derived from both membrane and intracellular proteins in a certain MHC context. Some studies point to advantageous low antigen density required for inducing TCR-T cytotoxicity as compared to CAR-T therapy (a few molecules versus hundreds or thousands, respectively) (29, 30); however, there are also observations based on high-resolution microscopy potentially establishing new sensitivity thresholds for CAR-T activation (31). Due to its high avidity, TCR-T does not require high affinity for activation, which may result in the scanning and sequential elimination of several antigen-expressing cells.

The first clinical success was achieved with TCR-T specific to the tumor-associated MAGE-A4 antigen in the HLA−A*02 context: Tecelra. It gained accelerated approval by the FDA in August 2024 for the treatment of synovial sarcoma on the basis of a high ORR rate and durable responses. According to published clinical reports, TCR-T therapeutics are also promising in melanoma (32, 33), multiple myeloma (34), HPV-driven cancers (35), and pancreatic (36) and liver cancers (33). So-called *public neoantigens* resemble an excellent class of TCR-T target because they are expressed only in tumor cells, in many patients, are highly homogenous, and may possess oncodriver activity, thus decreasing the probability of antigen loss. KRASG12 mutants are currently the best-validated neoantigens for TCR-T therapy (37–39).

At present, CAR-T and TCR-T therapeutics share the limitation of manufacturing complexity, and methods of *in vivo* transduction are urgently needed. One promising approach to overcoming the limitations of allogeneic CAR-T therapy is the leveraging of viral immune evasion mechanisms. Perica et al. (40) have demonstrated that the HIV-1 Nef protein enhances the persistence and efficacy of allogeneic CAR-Ts by reducing HLA-I expression to an optimal level, preventing both CD8^+^ T-cell–mediated rejection and NK cell activation. Additionally, Nef launches the Pak2 kinase pathway, protecting CAR-Ts from activation-induced cell death and enhancing their survival upon repeated antigen stimulation. This strategy represents a significant advancement against immune evasion for engineered T-cell therapies and potentially improves their applicability to off-the-shelf treatments.

### The engaging strategy

Another strategy for redirecting immune cells against malignant or autoreactive targets is the so-called *engaging strategy*. To overcome the restriction of natural TCR specificity, antibody- and TCR-based bispecific molecules have been developed. The essence of this strategy lies in bispecific antibodies, which possess dual specificity: one arm recognizing a tumor-associated antigen (TAA) and the other typically targeting CD3 (the invariant signaling component of the TCR). This dual targeting facilitates the formation of an immune synapse by physically bridging the T cell and the tumor cell. CD3 engagement is widely employed due to its invariance and constitutive expression on mature T cells, as well as T cell activatory function (41). Although this approach ensures broad T-cell activation, it may not represent the optimal strategy for selective immune engagement, as discussed below.

The second target can be any TAA most clearly represented in a given cancer type. This mode of action results in serial tumor cell killing, efficient even at picomolar concentrations of a BiTE and a low effector-to-target ratio. Blinatumomab (Blincyto, Amgen)—an anti-CD19 BiTE approved by the FDA in 2014 (1) for the treatment of B-cell acute lymphoblastic leukemia—was the first success in this field. In 2022–2024, there was a slurry of BiTEs approvals, with seven novel therapeutics entering the US market. The spectrum of targeted antigens includes CD20 (mosunetuzumab, glofitamab, and epcoritamab) and BCMA (elranatamab and teclistamab) (42), GPRC5D (talquetamab-tgvs), and DLL3 (tarlatamab). Tarlatamab has become the first in the T-cell engager (TCE) class to be used against solid tumors. It was approved for small-cell lung cancer patients with disease progression on chemotherapy and showed an ORR up to 40%, median progression-free survival of 5 months, and excellent intracranial control in patients with brain metastases (DCR 94%) (43). In contrast to blinatumomab—which is based on scFv format with extremely short half-life (2 h) and needs continuous infusion for 28 days and thus is very inconvenient for patients’ follow-up—recently approved BiTEs all involve full IgG-based design allowing for once-a-week subcutaneous dosing.

BiTEs have greatly changed the therapeutic landscape in hematological cancers. These drugs have response rates up to 90% and complete remission (CR) rates as high as 60% in relapsed/refractory B-cell lymphomas; such rates are impossible to reach with chemotherapy (44). In multiple myeloma, response rates of more than 60% and CR rates of nearly 40% are achieved in relapsed/refractory cases with more than three lines of previous treatment: truly incredible progress apparently (45).

## Clinical properties of TCEs

### Flexibility and independence from costimulatory signaling

One of the key advantages of antibody-based BiTE technology is its *independence* from MHC restriction and from costimulatory signals. The loss of MHC molecular complexes on tumor cells (as in the case of various viral infections) is one of the main causes of resistance to immunotherapy, including immune-checkpoint inhibition. In addition to antigen presentation, costimulation is also required for effective activation of T-cell immunity, and this state of affairs also complicates the machinery by increasing the number of targets that should be used to disrupt the holistic immune-response cascade. Simplification of immune-synapse formation by getting rid of all the “extraneous” factors is an aim of current TCE treatments. The formation of an immune synapse between a T cell and target cell, serial cell killing, and induction of perforin and granzyme release are mode-of-action characteristics shared by CAR-Ts and BiTEs. Furthermore, BiTEs and CAR-Ts act via both CD8^+^ and CD4^+^ T cells, though optimal differentiation stages differ, with Tem being the predominant engaged population in the case of BiTEs (46). The results of the BCMAxCD3 TCE study in multiple myeloma patients showed that the TCE response was driven by CD8^+^CX3CR1^+^ effector cells, being accompanied by both clonal expansion and effector differentiation; although the BCMA · CD3-T interaction was independent of MHC recognition, MHC class I–mediated signaling was required for activation of initially naïve cells (47).

### The toxicity profile and mitigation strategies

Due to predicted pharmacokinetics, TCEs appear to be a more controlled therapy in terms of on-target tumor toxicity as compared to cell-based treatments (CAR-T and TCR-T). Nonetheless, the two therapeutic modalities (TCE and CAR-T) share high toxicity including CRS and immune-effector-cell–associated neurotoxicity syndrome (48). In a phase 2/3 study (NCT01207388) of blinatumomab, grade ≥3 adverse events were seen in 71% of patients, with grade 3 neurological events in 24% (49). In the case of vibecotamab (Phase II clinical trials NCT05285813) (CD123/CD3), 59.2% of acute myeloid leukemia patients experienced CRS, which was the most common adverse event (50). A step-up dosing schedule is used to reduce the incidence and severity of CRS. This condition is thought to result from pan-T-cell provocation via CD3 binding. One way to overcome this limitation is to redirect some subpopulations of T cells. An elegant solution was suggested by Schmittnaegel with colleagues in 2015. The fusion molecule included an IgG whole antibody linked with a CMV-specific HLA complex, thus engaging an already pretrained subset of CD8^+^ T cells. Alternatively, engaging of T cells via coreceptor CD8 (unlike common CD3) is being employed in the latest trials (51, 52). T cells engaging via the TCR or both TCR and CD8 have also been shown to be relevant strategies, likely representing safe approaches with good activation potential (53). Currently, a dual anti-CD8/anti-TCR targeting TCE is being evaluated in a clinical trial (NCT06542250, primary completion 02.2028).

Another subpopulation that can be specifically engaged is mucosa-associated invariant T cells. These are an innate subset of T cells that express a common Vα7.2 chain. This approach is still in preclinical development, but there is already proof of concept available. The same barriers in the TME that hinder CAR-T activity in solid tumors, such as inadequate T-cell migration, T-cell exhaustion, and suppressive immune-cell populations, also apply to BiTEs. This situation limits their effectiveness. Unlike CAR-Ts, TCEs do not rely on active migration to reach a tumor site. This means that they can accumulate in tumors if CAR-T migration is impaired.

### Efficacy and multitargeting

TCEs hold promise for the treatment of solid tumors, despite challenges such as the immunosuppressive TME, antigen heterogeneity, and immune evasion. Advances in molecular engineering are enabling the creation of more durable and tumor-penetrating TCEs with enhanced specificity. To address antigen loss, multispecific engagers targeting multiple TAAs are being explored, reducing the risk of immune escape. Additionally, extending the half-life and stability of TCEs helps sustain therapeutic efficacy while minimizing systemic toxicity.

Emerging technologies such as half-life–extended TCEs (54), checkpoint-inhibitory TCEs (55), and simultaneous multiple-interaction TCEs (56) offer innovative ways to refine TCE selectivity and activity. These techniques enhance tumor targeting while mitigating off-target effects and improving both safety and efficacy. A detailed discussion of these strategies and their potential to revolutionize TCE-based immunotherapy will be presented in the following section.

Comparison of engagers with cell-based alternatives by important clinical factors are presented in summary **Table 2**.

**Table 2:** A comparison of TCEs with cell-based immunotherapies.

Therapy	Advantages	Limitations
**TCE**	- Broad antigen range using soluble TCR (3) - Solid tumor efficacy (57) - Escape adaptation (56) - Persistence (54) - ‘Off-the-shelf’ mode of action with relatively simple production process (46)	- Toxicity (48)
**CAR-T**	- Persistence (40)	- Toxicity - Antigen escape - Solid tumor infiltration - Manufacturing complexity (58) [Sterner and Sterner (59)]
**TCR-T**	- Broad antigen range including intracellular oncoproteins (60) - Solid tumor efficacy (61)	- Low persistence under optimization by overexpressing c-Jun (62) - Toxicity (61) - Antigen escape (35) - Manufacturing complexity (63)
**TILs**	- Solid tumor efficacy (8) - Broad antigen range due to polyclonal nature (6) - Low toxicity due to the lack of genetic modifications (64) - Escape adaptation due to polyclonal nature (65)	- Low persistence under optimization in combination with immune checkpoint blockade to augment the lifespan (66) - Manufacturing complexity under optimization using CliniMACS Prodigy^®^ Tumor Reactive T cell (TRT) Process (67)

TCE, T-cell engager; CAR-T, Chimeric antigen receptor T cell; TCR-T, T cell receptor T cell; TILs, Tumor infiltrating lymphocytes.

## Optimization of immune-cell engagers

### Antibody-based immune-cell engagers

**Figure 2:**
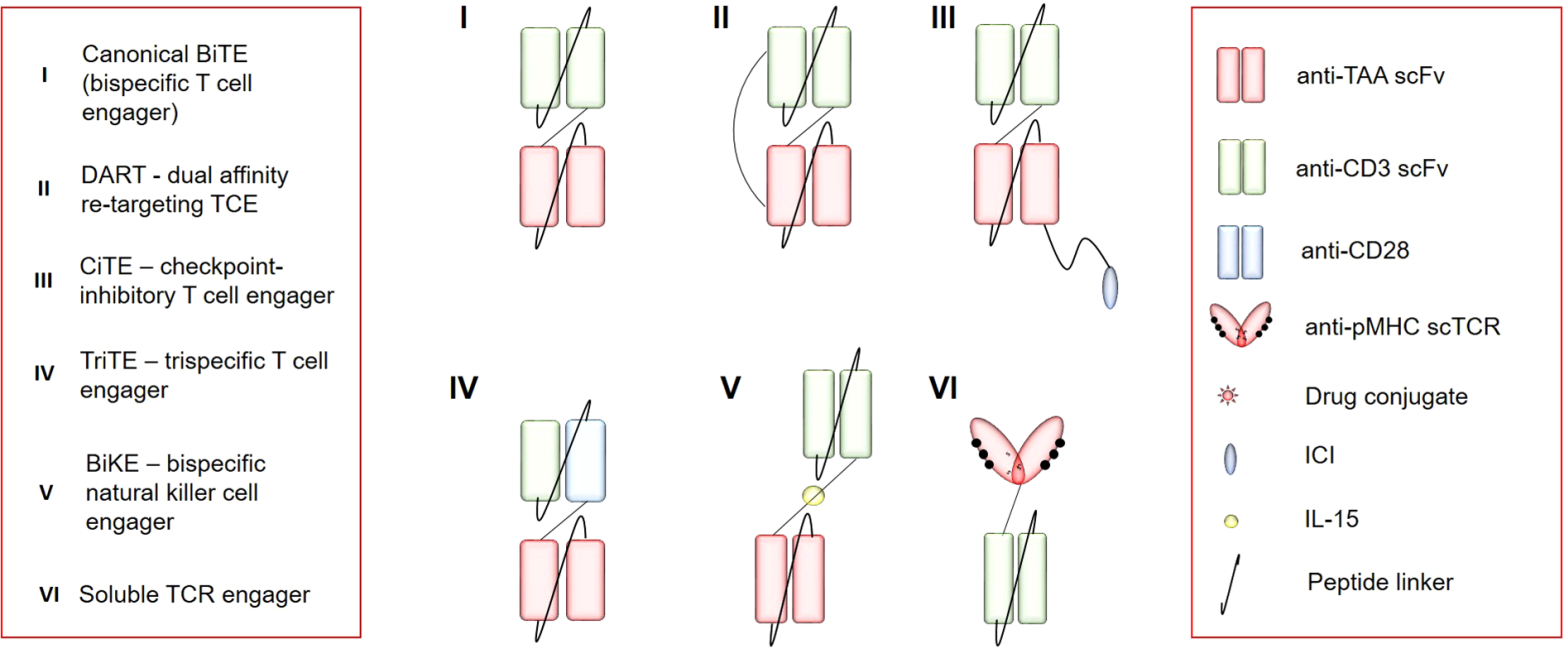
The repertoire of immune-cell engagers. TAA, tumor-associated antigen; scFv, single-chain variable fragment; CD3, cluster of differentiation 3; pMHC, peptide+major histocompatibility complex; scTCR, single-chain T-cell receptor; CD28, cluster of differentiation 28; ICI, immune-checkpoint inhibitor.

To date, clinical development of TCEs has been mainly focused on hematological cancers because they are good targets with high homogenous expression and lineage restriction. Selection of an appropriate antigen poses the main challenge for TCE application to solid tumors because there is a problem of tumor heterogeneity and antigen sharing with normal tissues. Solitomab (aEpCAM\aCD3) is an example of clinical failure due to the absent therapeutic window because the expression of epithelial cell adhesion molecule (EpCAM) on normal intestinal epithelia has led to dose-limiting toxicity and even fatal toxicity (68). Other features of solid tumors that greatly restrict TCE efficacy include low T-cell infiltration, anergy, and exhaustion due the immunosuppressive TME. Some of these limitations may be overcome by TCE optimization, including additional antigen specificities and functional modules. There are different BiTE modifications suitable for different classes and numbers of targets, and there is a constant search for stabler molecules with increased affinity. Be that as it may, there is an open field for experiments with different functional structures of canonical BiTEs (**Figure 2**).

Dual-affinity retargeting antibodies (DARTs) have cruciform structure that gives them greater stability. In preclinical testing, DARTs have shown more cytotoxicity as compared to BiTEs (69). PF-03732010, a P-cadherin specific DART, has been effective in several tumor models by inducing full regression of established tumors (70). Two DARTs linked by disulfide bonds—thereby maintaining the avidity of bivalent antibodies—form the structure of tandem diabodies. With the molecular weight of >100 kDa, they possess longer half-life and hence a pharmacokinetic advantage as compared to smaller antibody constructs. Tetravalent bispecific antibody AFM11 evokes effective T-cell activation as well as cytokine production in B-cell cancer models, in particular non-Hodgkin’s lymphoma, with affinity values 10 times higher than those of BiTEs (71). Anti-EGFRvIII tandem diabodies have been investigated in glioma models and show superiority to anti-EGFR despite 10–100-fold lower doses (72).

One of the strategies utilized to overcome an intratumoral suppressive microenvironment is to combine BiTE technology with immune-checkpoint blockade (73). This notion has been a rationale for the creation of bifunctional checkpoint-inhibitory TCEs, which in addition to the T-cell redirection module contain a variable domain against a certain immune checkpoint, such as PD-1 (55). A checkpoint-inhibitory TCE that simultaneously binds CD3 and PD-L1 activates T cells as well as NKT cells despite the presence of myeloid-derived suppressor cells and extends the survival of mice bearing established spontaneously metastatic melanoma xenografts (74). Another approach is to develop a BiTE for targeting T-cell coactivators. A trimeric EGFR/4-1BB BiTE has been constructed based on a nanobody platform and has shown antitumor activity with no systemic toxicity characteristic of agonistic 4-1BB antibodies (75).

A valid way to minimize on-target off-tumor toxicity of BiTEs is to restrict their activity to tumor sites by local expression. This may be achieved by combination of a TCE with CAR-T and OV platforms. As revealed in preclinical models, a TCE produced by CAR-Ts acts locally at the site where the cognate antigen is engaged by the CAR-Ts (76). EGFRvIII-specific CAR-Ts designed to secrete a T-cell–engaging antibody molecules (TEAMs) recognizing wild-type EGFR (CARv3-TEAM-E) have been evaluated in patients with recurrent glioblastoma (77). The combination of two specificities helped there to overcome glioblastoma’s heterogeneity in EGFRvIII expression, thereby leading to dramatic radiographic responses in three out of three participants within days after a single intraventricular infusion. In one patient, the effect was durable, lasting more than 150 days. The secretion of the anti-EGFR TCE by CAR-Ts was safe despite widespread expression of the target among tissues, though it is important to confirm the safety of this approach with systemic delivery of CAR-Ts (77). Another way to reduce unwanted toxicity is the targeting of surface coreceptor CD8 (instead of CD3) to engage only the cytotoxic subset of T cells but not the entire arsenal (78). On the other hand, efficiency in this case is supposed to be compromised by safety.

OVs offer another promising avenue for localized TCE expression, thus mitigating systemic toxicity while enhancing T-cell recruitment and activation at a tumor site. Engineered OVs have been successfully used as a platform for TCE delivery, thereby leading to a sustained immune response in preclinical models (79). For instance, an oncolytic adenovirus encoding a B7H3-targeting BiTE has manifested significant antitumor efficacy by enhancing T-cell–mediated tumor killing while reducing systemic exposure (80). This OV-mediated BiTE secretion method creates an immunostimulatory microenvironment, effectively redirecting virus-specific T cells toward tumors and overcoming tumor-induced immunosuppression (80). Such strategies highlight good potential of OV–TCE combinations to improve tumor-specific immune responses while maintaining safety. Moreover, there is another way to combine OVs’ potency with an “off-the-shelf” immunotherapy. Taha with colleagues have developed an elegant technology based on a dual-virus approach to overexpression of HER2T (truncated TAA HER2) on tumor cells. One component of this dual system (VSVΔ51) delivers a synthetic HER2 target to tumors, making them susceptible to an ADC called T-DM1, while the other virus (VV) expresses a HER2-targeted TCE to redirect T cells to attack the tumor. This approach overcomes limitations of target antigen absence in tumors, thus enhancing the efficacy of targeted immunotherapies (81).

Intensive research is directed toward increasing the number of targets of synthetic antibodies while maintaining affinity. This approach is related to another “trick” of cancer cells: in the process of malignant transformation, they acquire completely unconventional properties. In particular, antigen loss is one of such tricks (82). This process may be based on antigenic drift or a change in transmembrane trafficking of a certain surface antigen, similarly to what normally occurs with TCRs and the MHC complex during immune-synapse formation (83). In this regard, analogs of bispecifics are helpful, including simultaneous multiple-interaction TCEs capable of recognizing several targets at once in the event that some of them are “taken away” by a tumor cell (56).

Antitumor efficacy may be achieved not only by direct elimination of malignant cells but also by redirecting T-cell toxicity to cancer-supporting tumor-associated macrophages. Bi- and tri-specific TCEs recognizing folate receptor β (FRβ) expressed on M2-like macrophages have been studied in a model of malignant ascites. A shift toward a proinflammatory macrophage phenotype was observed there with increased expression of CD80 and CD86, and the percentage of residual CD11b^+^CD64^+^ cells diminished to an average of 37.9% (84). Regarding macrophages that resemble the predominant immune population in solid tumors and have strong tumor-promoting activities, targeting them may be a relevant strategy for altering the total TME and for achieving antitumor effects.

An important clinical limitation of prototypical BiTEs is their short half-life and the need for continuous intravenous administration. Therefore, currently, so-called half-life–extended BiTEs are being designed (54). Many of these are equipped with an additional Fc domain, creating bispecific antibodies with greater molecular weight. The Fc fragment may attract components of innate immunity (NK cells and macrophages), and therefore this class of synthetic molecules is functionally related to so-called bispecific natural killer cell engagers (85). Catumaxomab (EpCAM/CD3) was the first bispecific trifunctional antibody that reached the market; it was approved by the European Medicines Agency in 2009 for the treatment of malignant ascites. IgG-based bispecific antibodies have longer half-life *in vivo* as compared with scFv-based TCEs and also possess improved solubility and stability. At the same time, Fc inclusion may result in a nonspecific cytokine release and additional toxic effects due to binding to an array of Fcγ receptors on a variety of immune cells. Accordingly, the ADAPTIR platform has been engineered; it incorporates scFv homodimers in opposite orientations and Fc that is unable to bind FcγRs. Due to higher avidity, ADAPTIR-TCE has EC_50_ values approximately 30-fold lower than those observed with the tandem scFv of the same specificity. Diminished CD4^+^ T-cell vs CD8^+^ T-cell activation and a low level of a cytokine release have been demonstrated (86). Despite promising preclinical data (86), clinical development of an ADAPTIR-based BiTE (APVO414, PSMA/CD3) has been halted due to high immunogenicity and an unacceptable level of antidrug antibodies (87).

Designing engagers with an albumin-binding domain is also a feasible approach to half-life improvement. Trispecific T-cell–activating constructs (TriTACs) have three domains, binding to a tumor antigen, human serum albumin, and CD3ϵ (57). The domains binding a tumor antigen and albumin typically are single-domain antibodies (VHH). TriTACs possess a redirected lysis activity equivalent to that of BiTEs and comparable *in vivo* antitumor efficacy. Despite low molecular weight of approximately 53 kDa, TriTACs are suitable for once-weekly dosing in humans (57). TriTAC compounds HPN-217 and HPN-328 are currently undergoing phase I/II clinical trials against myeloma (NCT04184050) and small-cell lung cancer (NCT04471727), respectively. An alternative way to improve pharmacokinetics has been devised by BioNTech. Lipid nanoparticle (LNP)-formulated RNA (RNA-LNP) encoding a T-cell–engaging antibody specific for claudin 6 has been assessed in mice and cynomolgus monkeys; RNA-LNP maintained therapeutic serum concentrations sufficient for once-a-week injection (88).

Antibody-based engagers are a valid method for elimination of malignant cells and immunosuppressive populations and provide broad opportunities for the targeting of surface antigens. On the other hand, this mode of recognition has substantial limitations due to strong off-tumor on-target toxic effects. The use of TCR-based engagers may be a more targeted approach because it allows for the recognition of specific point mutations in intracellular proteins.

### TCR-based TCEs

Antibody-based bispecifics have shown efficacy against hematological tumors; promising developments are implemented in the field of BiTE-based solid-tumor therapies, but the repertoire of antibody targets is limited to surface-expressed proteins. On the other hand, ~90% of the proteome is localized within the cell and thus is inaccessible to antibodies (89, 90). Recognition of intracellular antigens is possible in the context of their presentation by MHC complexes.

TCRs, whose natural target is a complex of MHC, which represents the “inner world” of the cell, are the most amenable candidates for the role of such molecules. MHC I molecules are normally expressed on all cells except for cases of masking of MHC I complexes by cancerous cells or cells affected by some viruses (91). Thus, genetic and protein engineers have had to obtain TCRs that would maximally resemble antibodies, taking into account their strong affinity (on average three orders of magnitude stronger than that of TCRs) and soluble form.

First attempts to construct soluble TCRs consisting of only variable domains of both chains (Va+Vb) were not successful due to poor *solubility* and *stability* because of the high content of exposed hydrophobic residues normally shielded by a membrane (92). Nonetheless, researchers have encountered a number of technical challenges that stem from the very nature of the TCR, namely how to stabilize the chains in a soluble form while increasing avidity. At last, we currently have one FDA-approved BiTE based on a soluble TCR: tebentafusp (Kimmtrak^®^ from Immunocore), which is specific to HLA-A*02:01–positive TAA gp100 of uveal melanoma, which is one of the most common ocular malignant tumors (3). This biologic is related to the class of molecules called immune-mobilizing monoclonal TCR against cancer (ImmTAC). Currently, it is being evaluated in a phase II/III trial in nonocular melanoma (NCT05549297). The ImmTAC platform comprises an affinity-matured TCR fused to a humanized CD3-specific scFv. ImmTACs are highly efficient at killing tumor cells but have a half-life of only several hours and require frequent dosing. Other TCR-based TCEs in development target MAGE-A4/8, MAGE-A1, WT1 (93), or survivin (94).

A novel TCE concept has been proposed based on ectodomains of Vγ9δ2 T cells’ TCR as a tumor-targeting part, named gamma delta TCR anti-CD3 bispecific molecule (GAB). Vγ9δ2-TCR recognizes members of the butyrophilin (BTN) family; therefore, tumor targeting is independent of mutational load, MHC, and TAA expression. At present, the principal challenge for the GAB approach is the low expression level (95).

For example, a new class of engagers opens a huge opportunity for the targeting of potentially hidden intracellular antigens that remain undetected by conventional bispecific antibodies and CAR-T therapy. On the other hand, attempts to target oncopeptide–MHC complexes have been made with the use of the CAR-T platform: so-called peptide-centric CARs (96). Nonetheless, MHC remains a natural ligand for TCRs with all their features and optimal responsiveness. The only issue to be resolved is the transmembrane state of TCRs and their relatively weak affinity *in vivo*. Progress in genetic engineering techniques has given new opportunities for improving natural analogs of biological molecules, and TCEs are no exception.

## Engagers in autoimmunity

Collaboration of B and T cells lies at the core of chronic inflammation in autoimmune disorders (AIDs), and current therapeutic strategies are aimed at elimination of B- or T-cell subpopulations and at disruption of their activation and cross-talk (97). T and B cells interact through direct contact-dependent mechanisms and via secretion of cytokines and other soluble factors. To date, B–T-cell costimulation has been targeted through various pathways such as anti-CD52 (alemtuzumab) and CTLA-4–Fc fusion (abatacept). Alemtuzumab has been approved for multiple sclerosis (MS) treatment and shows insufficient efficacy in other AIDs; the use of alemtuzumab is also limited due to the safety profile: a black box warning of fatal autoimmune complications and cancer risk. Abatacept indications are broader including rheumatoid arthritis (RA), juvenile idiopathic arthritis, psoriatic arthritis, and the prophylaxis of acute graft-versus-host disease.

Targeting of inflammatory cytokines, their receptors, or downstream signaling leads to tremendous clinical success in a number of AIDs (RA, ankylosing spondylitis, Crohn’s disease, ulcerative colitis, psoriasis, psoriatic arthritis, and others) (98–101). Cytokines such as IL-12, IL-17, and TNF have pleiotropic activities guiding proliferation and activation of various populations of cells of adaptive and innate immunity, important for the control of infections. Because of broad immunosuppressive action, a prolonged cytokine block may entail a risk of serious infections and latent pathogen reactivation (102, 103). Precise targeting of T-cell and B-cell populations is a promising therapeutic tool for activation-induced cytidine deaminase upregulation as a safer alternative to anticytokine therapy and to generalized immunosuppression. Antibodies, CAR-Ts, and TCEs can be exploited in several areas of autoimmunity immunotherapy, where elimination of a specific T- or B-cell subset is a likely therapeutic goal. The existing approaches include bulk B-cell depletion (BCD), V or C region–targeted depletion of a T-cell subpopulation, and depletion of antigen-specific T or B cells.

Disease-modifying therapies targeting T- and B-cell populations have become mainstream in the treatment of MS. Those include recently approved anti-CD20 antibodies (ocrelizumab, ofatumumab, and ublituximab) (104) and rituximab used off-label. CD20 is a general B-cell marker expressed by the majority of B cells (starting from late pre-B lymphocytes) and is absent on terminally differentiated plasmablasts (PBs) and plasma cells (PCs). The absence of CD20 on fully mature PCs enables it to maintain humoral immunity against previously encountered pathogens despite anti-CD20 treatment. CD20 is also found on a subset of T cells with immunomodulatory and proinflammatory activities (105); these cells are enriched within the blood and cerebrospinal fluid of patients with MS. According to clinical data, an anti-CD20 treatment induces almost complete depletion of B cells and CD3^+^CD20^+^ T cells in circulation by week 2 after the first dose, and the repletion is slow, with a median of 26 weeks (ofatumumab) to 72 weeks (ocrelizumab) (104). Evaluation of BCD in tissues is a challenge, and incomplete B-cell clearance may lead to resistance. Low central nervous system (CNS) bioavailability of antibodies and inability to reach meningeal ectopic lymphoid follicles and subpial sites of inflammation are considered the main reason for anti-CD20 inefficacy at preventing long-term disability in secondary progressive MS (104). According to ofatumumab kinetics in cynomolgus monkeys, marginal-zone B cells in the spleen and lymph nodes are spared too. It can be theorized that due to the small size and to differences in pharmacokinetics and in the mechanism of action, TCEs may ensure more complete elimination of pathogenic B-cell populations in tissues. This idea is supported by data from oncological studies suggesting that CAR-Ts and TCEs may be active in the case of antibody resistance. A TCE can efficiently deplete B cells in the spleen and lymph nodes, as proven for an anti-CD20 TCE (106), thus overcoming one of mechanisms of resistance to BCD in AIDs. An important point is the use of T lymphocytes as effector cells to deplete B cells by means of CAR-Ts and a TCE instead of antibodies that rely on macrophages and NK cells as effectors. Owing to active migration of T cells, CNS bioavailability may be also improved. Blinatumomab’s activity against acute lymphoblastic leukemia with CNS infiltration is indirect proof of CNS bioavailability (107), indicating that a B-cell–depleting TCE may be promising for MS treatment. Nonetheless, an important point to consider with CD20-targeted therapeutics is the absence of CD20 on long-lived populations of autoantibody-producing PCs and PBs. This may be the reason for anti-CD20 resistance in a large proportion of patients with RA and immune thrombocytopenia (97) as well as for limited duration of the treatment response in patients with myasthenia gravis (108). Another problem is connected with internalization of antibodies; this drawback restricts the activity of antibody-dependent cellular cytotoxicity.

In contrast to CD20, B cells express CD19 at an earlier stage in development and retain this expression throughout all differentiation stages up to CD19^+^CD20^−^ PBs and some CD19^+^CD20^−^ PCs. CD19 regulates B-cell activation and is overexpressed in AIDs, suggesting that this biomolecule may be a valuable target. Current CD19-directed strategies include anti-CD19 monoclonal antibodies and CD19-targeted CAR-Ts and TCEs. The anti-CD19 monoclonal antibody inebilizumab is approved for the treatment of neuromyelitis optica spectrum disorder and has exerted a sustained effect on relapse risk and disability scores (109). In 2024, positive results of a phase III trial in immunoglobulin G4-related disease (IgG4-RD) were announced. The success of CAR-T therapy against B-cell cancers has inspired studies on CAR-Ts in a range of AIDs. Authors of initial clinical trials in systemic lupus erythematosus (SLE) have not reported severe cases of CRS-related toxicity, thereby showing promising efficacy results (110). Currently, 11 clinical studies on anti-CD19 CAR-Ts in SLE are active. Single-case studies of anti-CD19 CAR-T therapy indicate its good potential in antisynthetase syndrome (111), systemic sclerosis (112), idiopathic inflammatory myositis (112), and type 1 diabetes mellitus (113).

In the case of an AID, we must consider immunosuppressive conditions and consequent underlying effects including comorbid states. Thus, the application of CAR-T therapy may be complicated or even impossible due to the need for leukapheresis. Although there are successful cases of this protocol (114), alternatives are necessary. This and other limitations of CARs, such as uncontrolled *in vivo* administration, cumbersome manufacturing, and the high cost, may be to some extent overcome by the T-cell engagement approach. TCEs are much easier to produce as compared to CAR-Ts and may be more effective in the case of a low antigen level; in a direct comparison, TCEs outperform cytotoxic antibodies on target cell elimination (115). These features make TCEs a good therapeutic alternative. B-cell–depleting TCEs, just as CAR-Ts, are expected to be safer in AIDs in comparison to cancers, owing to the lower antigen load and reduced cytokine production (116). This notion is supported by the latest clinical data on SLE (110) and RA (117).

Clinical proof-of-concept data on a TCE in relation to AIDs have been obtained in refractory RA. In the latest study (117), six patients with multidrug-resistant severe RA received low-dose short-duration treatment with blinatumomab. Impressive efficacy was observed in all patients (including those resistant to previous BCD therapy): a decrease in disease activity scores, in synovial inflammation, and in levels of RA-associated autoantibodies. A reset of the B-cell profile was documented, with depletion of activated memory B cells and their replacement by non–class-switched IgD-positive naïve B cells.

In fact, autoreactive B cells in AIDs may constitute only 0.1–0.5% of all circulating B cells or less; hence, there is a strong rationale for restricted autoantigen-specific BCD (118). Autoreactive-B-cell elimination using chimeric autoantibody receptor (CAAR) and TCE-based approaches has been described. T cells have been engineered carrying CAAR consisting of the pemphigus vulgaris autoantigen (desmoglein [Dsg] 3) fused to CD137-CD3ζ signaling domains (119). Dsg3 CAAR-Ts effectively depleted anti-Dsg3 Nalm-6 B cells *in vivo* with efficacy comparable to that of CART19, and there was no activity against BCR^−^ cells. Depending on affinity, binding kinetics, and a relative position of the epitope, circulating auto-Dsg3 antibodies may have opposite effects on CAAR-T efficacy and in some cases even promote CAAR-T activity and persistence. Bispecific autoantigen T-cell engagers (BiAATEs) create an immunological synapse between autoreactive B cells bearing surface Ig and T cells and ensure precise elimination of autoreactive B cells, thus being much safer than CD20- or CD19-targeted approaches. A BiAATE has been constructed that consists of a-CD3 scFv and an immunogenic domain of phospholipase A2 receptor (PLA2R), described as a primary nephritogenic antigen in membranous nephropathy (120, 121). The BiAATE eliminates anti-PLA2R–secreting B cells isolated from patients with membranous nephropathy. Treatment of immunized hCD3 mice reduces the anti-PLA2R titer by 40%, with the effect persisting over a month since the end of treatment, implying an *in vivo* reduction in the number of anti-PLA2R B cells. BiAATEs have not yet been evaluated in the clinic. An ability to bind both an autoantibody and memory autoantibody-producing B cells may be a limitation, because in the case of high antibody titers, this feature may lead to competition and inefficient depletion of B cells or require higher doses for efficacy. Furthermore, a higher autoantibody load may be associated with higher CRS risk, and this question should be addressed too. For this reason, a BiAATE based on a TCR specific for autoantigen–MHC complexes may be more promising. In this case, the pathogenic autoimmune response may be targeted at two levels: antigen presentation and CD4^+^ T helper–B cell interaction.

In contrast to BCD, pan-T-cell depletion has unacceptable toxicity; consequently, selective targeting of specific subpopulations is the only feasible option. Because all αβ T cells carry either the T-cell receptor constant beta chain 1 (TRBC1) or TRBC2 constant β-chain gene segment, these may be targeted selectively. Such selective depletion of approximately half of all T cells has been proposed as a therapy for T-cell cancers using cytotoxic antibodies (122), an ADC (123), or CAR-Ts (124). At present, there is limited evidence for therapeutic depletion of T-cell subpopulations in AIDs. Aside from CD20^+^CD3^+^ T-cell elimination in MS mentioned above, successful examples include T-cell receptor variable beta chain (TRBV)-directed therapy. VDJ recombination results in expression of 1 of the 30 TRBV gene families on the surface of each T cell. Accordingly, each TRBV variant is expressed on the surface of 1% to 5% of all peripheral-blood normal T cells; therefore, a therapy that involves V segment-based T-cell elimination allows a physician to precisely cut out small subpopulations of all T cells in a sort of liquid surgery. This methodology has been proposed for AIDs and mature-T-cell lymphomas (125–127). The TRBV9-containing CD8^+^ TCR motif is reported to be associated with the pathogenesis of ankylosing spondylitis, psoriatic arthritis, and acute anterior uveitis; cognate HLA-B*27–presented epitopes have been identified (128). The first anti-TRBV9 treatment of an ankylosing spondylitis patient was successful, resulting in >5-year CR (127). Preliminary phase II data show superiority of anti-TRBV9 (seniprutug) over historical data on adalimumab in terms of critical clinical parameters such as Assessment of SpondyloArthritis International Society (ASAS) 20 and ASAS40 (129). Seniprutug was approved in Russia in 2024 and is currently evaluated in two international studies (NCT05445076 and NCT06333210). A TRBV-specific TCE (130) and CAR-Ts (131) have been suggested for the treatment of T-cell cancers; they may also be a valuable option for AIDs featuring pathogenic TRBV-restricted T cells. A TRBV9-specific TCE was reported recently (132).

An interesting technology for elimination of autoreactive-T-cell clones has been proposed by Goldberg and colleagues. This approach exploits a soluble pMHC-II heterodimer fusion protein recognizing the DRB1*04:01-restricted CII_259 peptide from collagen II: an antigen described in RA. The pMHCs were biotinylated and conjugated with cytotoxic drug monomethyl auristatin F. After tetramerization, potent peptide-specific killing of a hybridoma cell line was achieved. In a more translatable therapeutic strategy, a 3DNA nanocarrier platform was utilized to improve biocompatibility. This pMHC-based engager manifested efficient rates of on-target cytotoxicity *in vitro* (4). Toxin-coupled MHC class I tetramers have been successfully used for specific elimination of IGRP^+^ CD8^+^ T cells in diabetic mice (133). Clinical validation of antigen-specific T-cell targeting in AIDs is eagerly awaited.

Despite the recent progress in AIDs’ treatments with new biologics and small-molecule compounds, there is still a substantial need for novel therapeutic approaches effective against mechanisms underlying AIDs. TCEs are likely a promising tool for AID treatment because of their efficacy in precise and deep elimination of pathogenic immune-cell populations.

## Conclusion

TCEs have evolved from hematology-focused BiTEs to sophisticated multispecific platforms capable of targeting solid tumors. Innovations in conditional activation, costimulation, and TCR-mediated intracellular antigen recognition address historical limitations of antibody-based therapies. Although toxicity management and antigen escape remain challenges, ongoing clinical trials testing combination regimens and novel targets are expected to improve TCEs’ therapeutic index. Future success will hinge on biomarker-driven patient stratification, advances in protein engineering, and synergistic integration with complementary immunomodulators. As the field progresses, TCEs are poised to become cornerstone therapies for diverse cancers.

TCEs may be a valuable tool for deep elimination of B-cell and T-cell subpopulations involved in the pathogenesis of AIDs. Preliminary clinical data indicate their efficacy in cases of resistance to antibody-mediated cell depletion. Development of TCE-based approaches for precise targeting of autoantigen-specific cells is a promising area of future research.
